# Open label study of escalating doses of oral treprostinil diethanolamine in patients with systemic sclerosis and digital ischemia: pharmacokinetics and correlation with digital perfusion

**DOI:** 10.1186/ar4216

**Published:** 2013-04-18

**Authors:** Ami A Shah, Elena Schiopu, Laura K Hummers, Michael Wade, Kristine Phillips, Cynthia Anderson, Robert Wise, Francesco Boin, James R Seibold, Fredrick Wigley, Kristan D Rollins

**Affiliations:** 1 Division of Rheumatology, Department of Medicine, Johns Hopkins University School of Medicine, 5501 Hopkins Bayview Circle, Room 1B7, Baltimore, MD 21224 USA; 2 Division of Rheumatology, Department of Internal Medicine, University of Michigan, Suite 7C27, North Ingalls Building, 300 North Ingalls Street, SPC 5422, Ann Arbor, MI 48109-5422 USA; 3 United Therapeutics Corp., 55 TW Alexander Drive, Research Triangle Park, NC 27709 USA; 4 Scleroderma Research Consultants LLC, 97 Deer Run, Avon, CT 06001 USA

## Abstract

**Introduction:**

Treprostinil diethanolamine is an innovative salt form of the prostacyclin analogue, treprostinil sodium, developed as an oral sustained release (SR) osmotic tablet. The availability of a formulation permitting convenient systemic delivery might have applicability to scleroderma vascular complications. We evaluated pharmacokinetics and perfusion in scleroderma patients with digital ischemia following escalating twice-daily doses of treprostinil diethanolamine SR.

**Methods:**

Scleroderma patients with digital ulcers were enrolled in this dual-center, open-label, phase I pharmacokinetic study. Drug concentrations and perfusion, quantified by laser Doppler imaging, were measured over 12 hours at the 2 mg and 4 mg (or maximally tolerated) doses. Pharmacokinetic parameters were determined from individual plasma concentration versus time profiles using non-compartmental analysis methods. Digital perfusion and skin temperature were modeled as a function of log-transformed drug concentration and other covariates by performing repeated measures analyses using random effects models.

**Results:**

Nineteen scleroderma patients (84% female, 53% limited scleroderma) received treprostinil diethanolamine SR with dose titration up to 4 mg twice daily as tolerated. Peak concentrations (mean maximum plasma concentration (C_max_) = 1,176 and 2,107 pg/mL) occurred approximately 3.6 hours after dose administration, and overall exposure (under the plasma concentration-time curve from time 0 to 12 hours post dose (AUC_0-12)_ = 7,187 and 12,992 hr*pg/mL) was linear between the 2 mg and 4 mg doses. Perfusion and digital skin temperature were positively associated with log-transformed plasma concentration at the 4 mg dose (*P* = 0.015 and *P* = 0.013, respectively). The most frequent adverse events were similar to those seen with prostacyclin analogues.

**Conclusions:**

Oral treprostinil diethanolamine was effectively absorbed in patients with scleroderma. Drug administration was temporally associated with improved cutaneous perfusion and temperature. Treprostinil diethanolamine may provide a new therapeutic option for Raynaud's phenomenon and the peripheral vascular disease of scleroderma.

**Trial Registration:**

ClinicalTrials.gov NCT00848939.

**Electronic supplementary material:**

The online version of this article (doi:10.1186/ar4216) contains supplementary material, which is available to authorized users.

## Introduction

Treprostinil is a chemically stable analog of prostacyclin. The major pharmacological actions of treprostinil are direct vasodilation of pulmonary and systemic arterial vascular beds, inhibition of platelet aggregation, and inhibition of vascular smooth muscle cell proliferation [1–4]. Treprostinil, as the sodium salt, has shown clinical effectiveness in the treatment of pulmonary arterial hypertension (PAH) when administered by the continuous subcutaneous and intravenous (via infusion pump) and inhaled (via nebulizer device) routes of administration as the approved products Remodulin Injection and Tyvaso^®^ Inhalation Solution, respectively [5]. Treprostinil diethanolamine is an innovative salt form developed for oral delivery as a sustained release (SR) osmotic tablet. Both treprostinil sodium and treprostinil diethanolamine disassociate in blood and exist as the ionized, bioactive form of treprostinil.

Patients with diseases, such as systemic sclerosis (scleroderma, SSc), which involve perturbation of the peripheral vasculature, experience abnormalities in the blood vessels and mal-distribution of blood flow away from the nutritive capillaries that supply the skin. Raynaud's phenomenon is an episodic vasoconstriction of small arteries in response to cold or stress that occurs in more than 90% of patients with SSc [6]. An associated small vessel obliterative vasculopathy frequently leads to the development of ischemic digital ulcers [7], a complication occurring in more than 40% of SSc patients [8]. As a chemically stable tricyclic benzindine analog of prostacyclin, treprostinil inhibits platelet aggregation, induces vasodilation, and suppresses smooth muscle proliferation. Improvement in microvascular arterial blood flow and velocity in lower limbs and reduction in ischemic pain and healing of digital ulcers and other ischemic wounds has been observed with infused prostacyclin and prostacyclin analogs [9–19]. As an orally available formulation, treprostinil diethanolamine SR avoids the need for infusion devices and the associated demands and risks associated with the intravenous or subcutaneous route of delivery and may provide a treatment option for the peripheral vascular disease seen in SSc.

The aim of the present study was to determine the pharmacokinetic, safety and tolerability profiles following escalating twice-daily doses of treprostinil diethanolamine SR in patients with SSc. During this pharmacokinetic trial, we also quantified changes in digital perfusion by laser Doppler imaging and correlated levels of perfusion with plasma drug concentration.

## Methods

### Study design

The study was a two-center, open-label, pharmacokinetic study in adult subjects with scleroderma who were enrolled at the Johns Hopkins Scleroderma Center and the University of Michigan Scleroderma Program at Ann Arbor (http://ClinicalTrials.gov Identifier: NCT00848939). Approval from the Johns Hopkins Medicine Institutional Review Board and the University of Michigan Medical School Institutional Review Board for the study and written informed consent from subjects was obtained prior to performing any study-related procedures. This study was generally conducted in accordance with the ethical principles that have their origins in the Declaration of Helsinki and the ICH GCP E6 Good Clinical Practice guidance.

### Inclusion criteria

Male and female subjects were eligible for participation in the study based on the following criteria: subjects were older than 18 years of age, met American College of Rheumatology criteria [20] for SSc, and had either an active digital ulcer or history of digital ulceration occurring within the past six months and at least six to ten Raynaud's phenomenon episodes per week. Conventional therapies for SSc and digital ulcers, including calcium channel blockers, were permitted at stable doses throughout the study.

### Exclusion criteria

Exclusion criteria included an established diagnosis of PAH, low body weight (less than 40 kg), concomitant other connective tissue disease, postural hypotension or syncope, anemia (hemoglobin less than 75% of lower limit of normal), moderate to severe hepatic impairment or abnormal transaminases (greater than three times the upper limit of normal), intractable diarrhea, severe malabsorption, severe organ failure, cigarette smoking within six months, pregnancy, or lactation. Additionally, subjects who had an upper limb sympathectomy in the last year, or received treatment with a parenteral prostanoid within the previous three months, local botulinum toxin injection within one month, or systemic antibiotics to treat infected digital ulcers within two weeks of the baseline visit were excluded. Subjects receiving treatment with a phosphodiesterase inhibitor therapy except for erectile dysfunction were also excluded. Use of gemfibrozil, cyclophosphamide, glitazones, rifampin and grapefruit-containing products was not permitted over the course of the study. The investigators recorded other comorbid conditions and organ system involvement that did not meet these exclusion criteria.

### Treatment

Treprostinil diethanolamine SR was provided as 0.25 mg, 0.5 mg and 1 mg strengths for administration in the study. Oral dosing of treprostinil diethanolamine SR was initiated at 0.25 mg twice daily (BID) and the dose escalated in 0.25 mg increments every 48 hours at the evening dose, based on tolerability, up to a target dose of 4 mg BID (or maximum dose tolerated by each subject at the end of the eight-week escalation period). Subjects were instructed on the appropriate combination of tablets to administer for each dose and to take doses approximately every 12 hours within 10 to 15 minutes following a morning and evening meal containing approximately 500 calories.

### Sample collection

Patients underwent 12-hour pharmacokinetic sampling upon reaching doses of 2 mg and 4 mg (or at the maximum achieved dose at the end of the titration period). Subjects who did not reach a dose of 2 mg BID during the study had assessments performed only at the end of study visit at their maximally tolerated dose. All pharmacokinetic blood sampling took place in the General Clinical Research Center (GCRC) and occurred after the subject had maintained the dose for at least five days. Subjects consumed a standardized breakfast (totaling approximately 500 calories) 10 to 15 minutes prior to the administration of treprostinil diethanolamine SR on pharmacokinetic sampling days.

Venous blood samples for pharmacokinetic assessments were obtained by venipuncture or in-dwelling cannula with a saline lock (no heparin). Eight venous blood samples of approximately 5 mL were collected in K_3_-ethylenediaminetetraacetic acid (EDTA) vacutainer tubes over the 12-hour dosing interval at the following time points: prior to dosing, and at 1, 2, 4, 6, 8, 10, and 12 hours post-dose for determination of treprostinil plasma concentrations.

### Bioanalytical parameters

Within one hour of blood collection, plasma was separated by centrifugation at 4°C for 10 to 15 minutes at 3,000 g, harvested and immediately stored frozen at -20 to -25°C until shipment on dry ice for analysis. Plasma samples were analyzed by Enthalpy Analytical, Inc. (Durham, NC, USA). Treprostinil concentrations were determined by a validated method using solid-phase extraction followed by ultra-performance liquid chromatography coupled with tandem mass spectrometry detection (HPLC/MS/MS) [21]. The lower limit of quantitation was 10 pg/mL using a 150 µL aliquot of human plasma, and the linear concentration range for the calibration curve was 10 to 5,000 pg/mL. The accuracy and precision were determined during validation by analyzing the quality control (QC) sample concentrations to ensure that the calibration standards that were analyzed were suitable for generating calibration curves. The acceptance criteria required the average accuracy be within ±15% and the precision should be ≤15% for low, mid, high, and dilution QC levels. For the lower limit of quantification QC level, the average accuracy needed to be within ±20% and the precision had to be ≤20%. Greater than or equal to half of the QC samples at any given concentration had to meet accuracy acceptance criteria. In addition, greater than or equal to two-thirds of all QC samples had to meet accuracy acceptance criteria.

### Pharmacokinetic analysis

Pharmacokinetic parameters were determined from individual plasma concentration versus time profiles using non-compartmental analysis methods and WinNonlin version 5.2.1 (Pharsight Corporation, Mountain View, CA, USA). Pharmacokinetic analysis included determination of the observed maximum plasma concentration (C_max_), time to maximum concentration (t_max_), area under the plasma concentration-time curve from time zero to the last time point with measurable drug concentration as calculated by the linear trapezoidal method (AUC_0-t_), area under the plasma concentration-time curve from time zero to 12 hours post dose (AUC_0-12_), and apparent terminal elimination half-life (t_1/2_). Pharmacokinetic parameters were summarized by calculating the mean, median and coefficient of variation (CV%) by dose regimen. Pharmacokinetic parameters derived from subjects' treprostinil concentration profiles at doses other than 2 mg BID or 4 mg BID were excluded from the statistical summary and comparisons. Actual sampling times were used in the calculations of pharmacokinetic parameters. Plasma concentrations that were below the lower limit of quantitation were set to zero when calculating summary statistics for plasma concentration at a given nominal time point, except when a single value fell between two measurable concentrations, then this value was set to be missing.

Dose proportionality in treprostinil AUC_0-12_ and C_max_ following repeat doses of 2 mg and 4 mg BID was assessed by analysis of covariance (ANCOVA). C_max_ and AUC_0-12_ were dose normalized (to 1 mg) and log_e_-transformed values were analyzed using a mixed-effect model by restricted maximum likelihood (REML) using SAS Proc Mixed, with dose regimen as a fixed effect and subject as a random effect. The least squares mean (LSM) difference in the parameter estimates between the two dose regimens and the 90% confidence interval (CI) for the difference were calculated. The difference and CI were then transformed back to the original scale to provide an estimate of the geometric LSM (GLSM) and 90% CI as the parameter ratio, 4 mg BID versus 2 mg BID.

### Measurement of digital perfusion and skin temperature

Subjects enrolled at the Johns Hopkins Scleroderma Center also had serial measurements of digital perfusion performed at each pharmacokinetic visit pre-dose and 1, 2, 4, 6, 8, 10 and 12 hours post-dose. Laser Doppler imaging was performed using the Moor Instruments LDI2-IR imaging system (Axminster, Devon UK). Perfusion was expressed as arbitrary perfusion units (PU). Laser Doppler imaging was performed in a quiet, temperature controlled room (20 to 23°C) following an acclimation period of 15 minutes prior to initiation of testing. Digital skin temperature was measured immediately prior to each laser Doppler imaging scan by affixing a thermistor (YSI, Yellow Springs, OH, USA) to the pad of the third distal phalanx of the hand under study.

Mean perfusion of the middle three fingers of both hands and mean digital skin temperatures at pre-dose trough (time 0) were compared to 12 hours post-dose for each pharmacokinetic visit using the paired t-test after the normality of these variables' distributions was confirmed by the Shapiro-Wilk test. Repeated measures analyses using random effects models were performed using perfusion and skin temperature as dependent variables of interest and perfusion obtained at drug trough, log-transformed drug concentration, visit number, hour of Doppler assessment, and left versus right hand as potential explanatory variables. Covariates with *P*-values <0.15 were retained in the statistical model. All data were used across time points, visits, and hands. AUC for the perfusion parameter was calculated using the trapezoidal rule on the observed perfusion values.

### Tolerability and safety

Throughout all treatment phases, vital signs, the occurrence of adverse events and use of concomitant medication was monitored for each subject. The rating of intensity of adverse events was by subject report and physician determination. Samples for clinical laboratory tests were collected prior to the screening visit and at study exit. Treatment-emergent clinically significant abnormalities in vital signs and clinical laboratory data were examined.

## Results

### Study population

A total of 20 patients with SSc (ten at each center) were enrolled between March and December 2009. Nineteen subjects received at least one dose of study drug, and 16 subjects completed the eight week study in its entirety. Four subjects discontinued the study prematurely. Two subjects discontinued due to adverse events, one subject was withdrawn due to protocol violation, and another subject was lost to follow-up after undergoing the baseline visit. Two of these subjects withdrew after the first pharmacokinetic visit, and two subjects withdrew prior to undergoing any pharmacokinetic assessments.

The population was predominately female (84.2%) and Caucasian (89.5%), with the limited cutaneous subset of SSc (52.6%). The subjects had ages ranging from 34 to 73 (mean: 48) years, body weights ranging from 46.4 to 95.1 (mean 69.3) kg, and body mass indices (BMI) ranging from 18.4 to 34.1 (mean 24.9) kg/m^2^. Eleven of the 19 subjects had an active digital ulcer at study entry. Baseline medication use included proton pump inhibitors (74%), calcium channel blockers (63%), aspirin (37%), selective serotonin uptake inhibitors (21%), ACE-inhibitors or angiotensin receptor blockers (16%) and statins (11%). Cilostazol, clopidogrel, and pentoxifylline each were used in 5% of subjects. No patient was on a nitrate or endothelin receptor antagonist. A summary of baseline characteristics for subjects who received at least one dose of study drug and constituted the analysis population is presented in Table 1.

**Table 1:** Summary of Baseline Characteristics.

Characteristic	Number = 19
Age in years, mean (SD)	48 (10)
Gender: Male/Female (number)	3/16
Race: Caucasian/African American (number)	17/2
Weight in kg, mean (SD)	69.3 (14.2)
BMI in kg/m^2^, mean (SD)	24.9 (4.7)
SSc subtype, number Limited Diffuse	10 9
Duration of disease in years, mean (SD)	11.5 (6.7)
Organ involvement, number Esophagus/Gastrointestinal Lung Fibrosis Renal Liver Gallbladder	16 6 1 1 3
Autoantibody status, number positive Anti-centromere^a^ Anti-topoisomerase 1^b^	7 3

^a^Number = 18 with available data; ^b^Number = 17 with available data. BMI, body mass index; SSc, systemic sclerosis.

### Pharmacokinetics

Seventeen subjects reached a 2 mg BID dose, and 12 subjects achieved the 4 mg BID dose and underwent pharmacokinetic assessments. At the end of the eight week dose escalation period, four subjects completed the study at a maximum tolerated dose less than 4 mg BID: one subject each completed the study at 1 mg BID, 2 mg BID, 2.5 mg BID and 0.5 mg BID. The overall average duration of exposure in the study was 47.7 days (range 15 to 62 days), with an overall mean of 21 and 42 days to reach the 2 mg BID and 4 mg BID doses levels, respectively.

**Figure 1:**
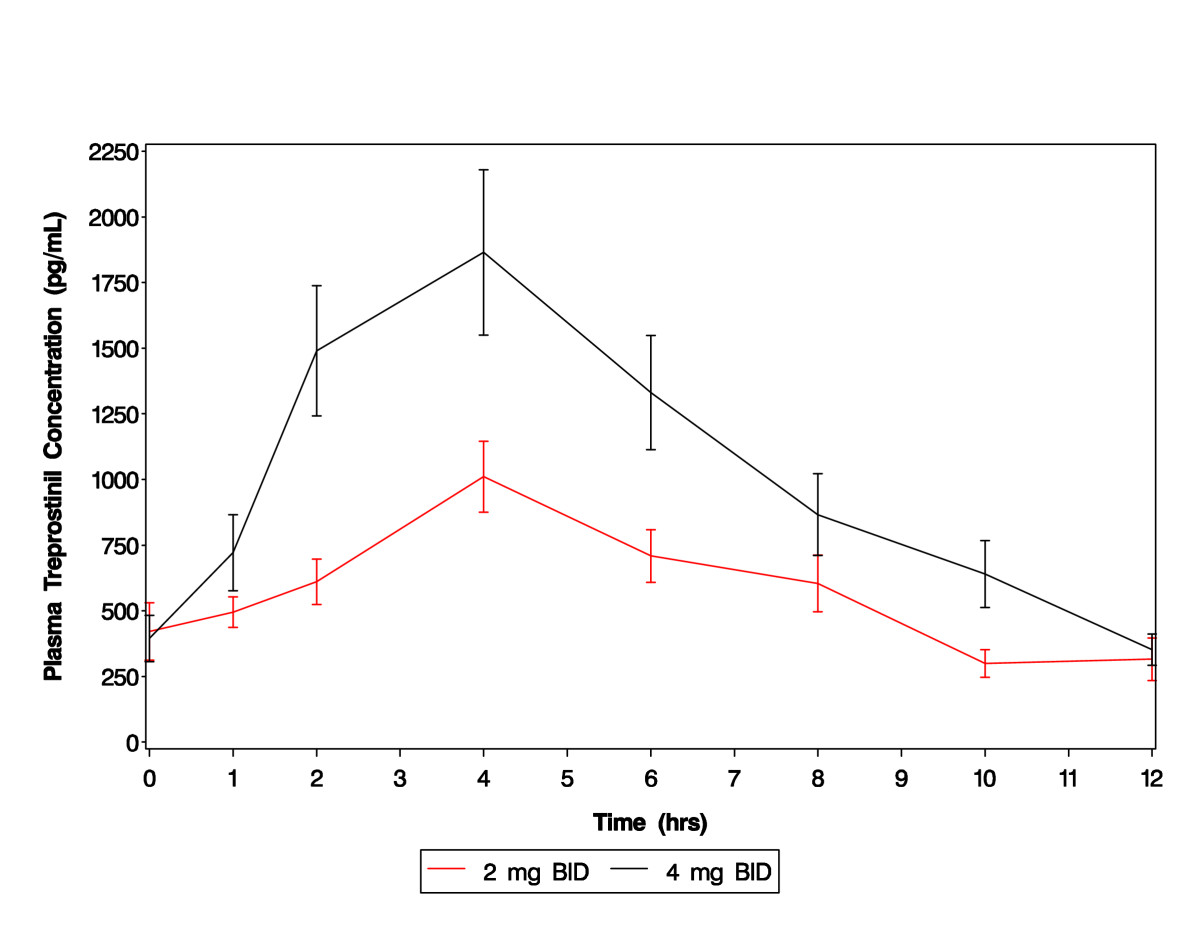
**Mean treprostinil plasma concentration versus time curves following repeat doses of 2 mg BID and 4 mg BID of treprostinil diethanolamine SR in SSc patients**. Dose-related increases in treprostinil concentrations were observed over the 0 to 12 hour post-dose period. Bars represent the standard error of the mean. BID, twice daily; SR, sustained release; SSc, systemic sclerosis.

Mean plasma treprostinil concentration versus time profiles following repeat doses of 2 mg BID and 4 mg BID regimens are depicted in Figure 1. The data demonstrate that treprostinil diethanolamine was rapidly absorbed following administration of repeat doses of SR tablets when given with a 500-calorie meal. All pre- and post-dose samples had measureable treprostinil plasma concentrations, and the mean concentrations in pre-dose and 12 hour post-dose samples were comparable for each BID regimen suggesting that an approximate steady state condition was achieved.

Pharmacokinetic parameter estimates are summarized in Table 2. Maximum plasma treprostinil concentrations generally occurred at a median of 4.0 hours after dose administration at both dose levels. The t_1/2_ of treprostinil following repeated oral SR doses was approximately 3.4 to 3.6 hours, and was similar in both dose levels and with that reported with the infused formulation. There was high inter-subject variability in all pharmacokinetic parameters studied.

**Table 2:** Pharmacokinetic parameters of treprostinil in SSc patients following dose escalation to 2 mg and 4 mg BID.

Cohort Regimen	Statistics	C_max_ (pg/mL)	t_max_ (hour)	AUC_(0-12)_ (hr*pg/mL)	t_½_ (hour)
**2 mg BID** (Number = 17)	GeoMean CV% Median Mean CV_b_% Minimum Maximum	1,063 51.1 1,190 1,176 43.6 428 2,200	na na 4.0 3.58 74.3 0.0 12.0	6,400 56.9 6,764 7,187 45.2 2027 13,820	2.94 61.6 3.28 3.39 56.0 1.07 8.48
**4 mg BID** (Number = 12)	GeoMean CV% Median Mean CV_b_% Minimum Maximum	1,895 52.8 1,930 2,107 46.3 752 3,950	na na 4.0 3.67 31.5 2.0 6.0	11,880 45.0 11,530 12,922 43.1 6726 24,150	3.19 55.8 2.88 3.62 55.1 1.74 7.75

BID, twice daily; CV%, coefficient of variation for geometric mean, CV_b_%, coefficient of variation for mean; GeoMean, geometric mean; na, not applicable; SSc, systemic sclerosis.

Ninety percent CIs for the GLSM ratios for dose-normalized AUC_0-12_ (ratio 1.10; 90% CI 0.97 to 1.25) and C_max_ (ratio 1.03; 90% CI 0.89 to 1.19) demonstrated dose proportionality between the 2 mg and 4 mg BID regimens.

### Digital perfusion and skin temperature

Of the 10 subjects enrolled at the Johns Hopkins Scleroderma Center, nine subjects underwent a laser Doppler imaging assessment upon reaching a 2 mg BID dose (first pharmacokinetic PK) visit). Pre-dose perfusion data were not available in one subject due to laser Doppler device malfunction. Eight subjects participated in laser Doppler imaging assessment at the end of study (second PK) visit, six subjects at the 4 mg BID dose and one subject each at the 0.5 mg BID and 1 mg BID doses, respectively. Average time on therapy at the first and second PK visits was 26 ± 4 days and 54 ± 4 days, respectively.

**Figure 2:**
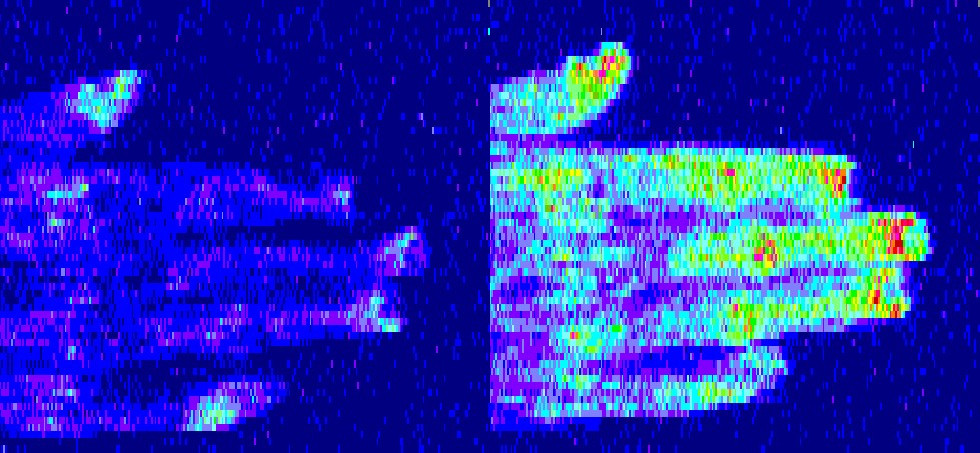
**Perfusion as assessed by LDI improved after medication administration**. Sample images from Subject 6 at the second PK/end of study visit are shown. The baseline image at drug trough prior to administration of a 4 mg dose (left) corresponds to a mean perfusion of 89.3 units and a skin temperature of 25°C. Twelve hours after dosing, perfusion has improved to 298.3 units with a skin temperature of 32°C (right). LDI, laser Doppler imaging; PK, pharmacokinetic.

**Figure 3:**
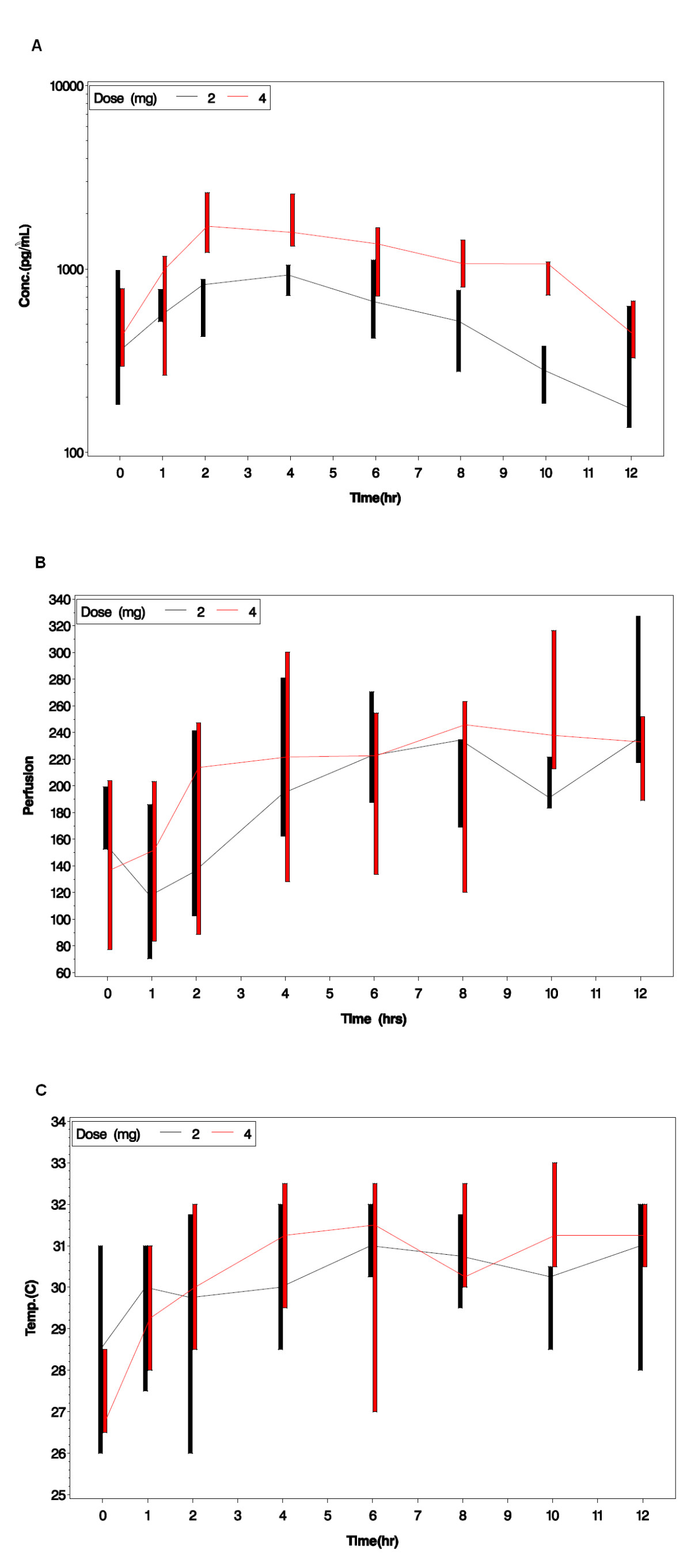
**Median drug concentration, perfusion, and skin temperature over time**. Graphs depict the median log-transformed treprostinil plasma concentration time profile (Panel **A**) in pg/mL for subjects reaching a 2 mg BID dose (N = 8) and 4 mg BID dose (N = 6) and corresponding changes in median skin perfusion in PU (Panel **B**) and skin temperature in ºC (Panel **C**) over time. Bars represent the interquartile (25th to 75th percentile) range. BID, twice daily; PU, perfusion units.

**Figure 4:**
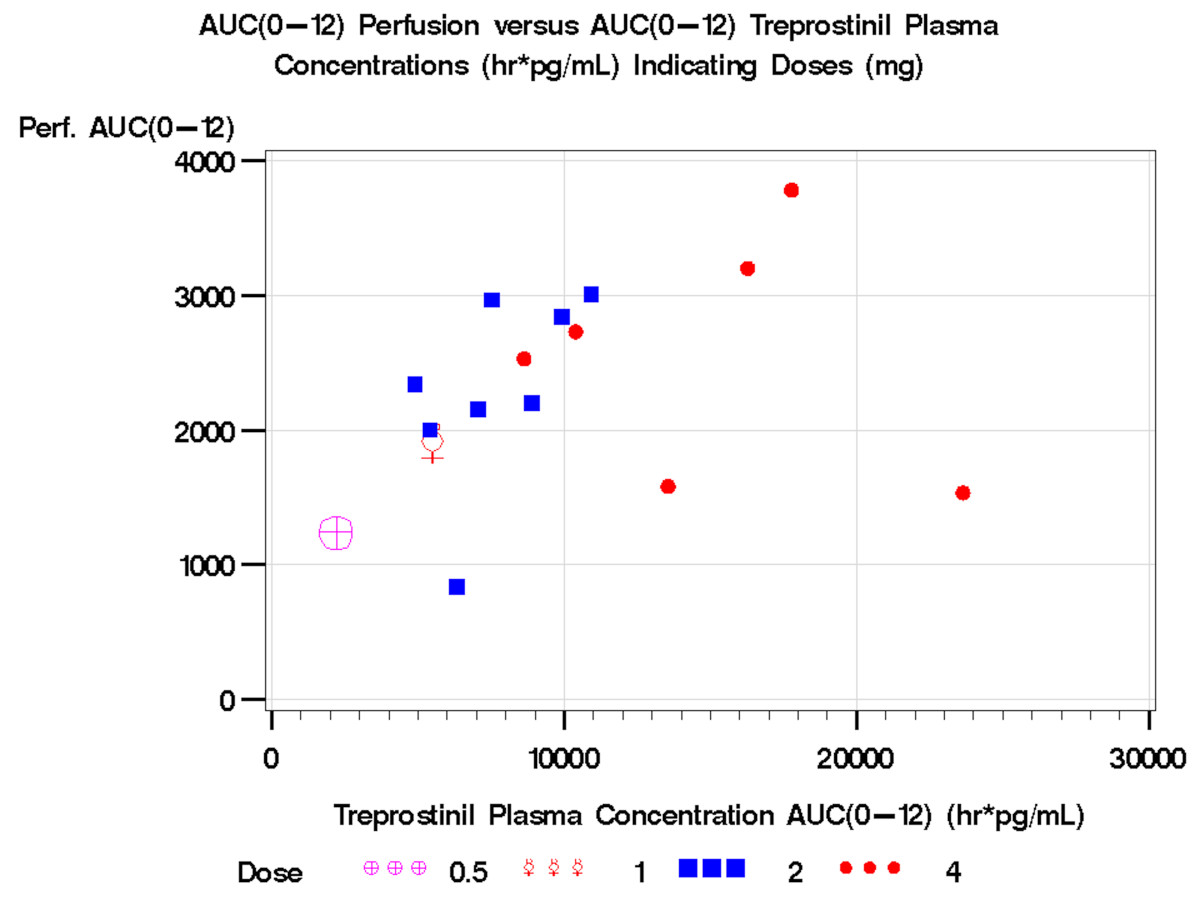
**Perfusion area under the curve (AUC) versus concentration AUC**. Increased plasma concentrations were observed with increases in treprostinil diethanolamine SR dose. An increase in perfusion was observed with an increase in drug exposure. SR, sustained release.

Mean perfusion increased over the 12-hour dosing interval at the first PK evaluation from a pre-dose trough of 175.4 PU (SD 59.7) to 244.3 PU (SD 66.0) at 12 hours (N = 8, *P* = 0.09). Correspondingly, the skin temperature increased from 28.5°C (SD 2.6) to 30.2°C (SD 2.9) (N = 8, *P* = 0.14). A significant increase in perfusion from the pre-dose trough over the 12-hour dosing interval (137.2 PU (SD 66.2) to 207.3 PU (SD 61.4); N = 8, *P* = 0.042) and digital skin temperature (27.3°C (SD 1.9) to 30.3°C (SD 2.4); N = 8, *P* = 0.018) was observed at the second PK/end of study evaluation. Significant improvements in perfusion were demonstrated after medication administration (Figure 2). Perfusion and digital skin temperature were positively associated with log-transformed plasma concentration at the second PK/end of study visit (*P* = 0.015 and *P* = 0.013, respectively), but not at the first PK visit, after adjusting for hour of Doppler assessment (Figure 3). The perfusion AUC_0-12_ at 2 mg was 2,295.88 PU (SD = 708.52; N = 8) and perfusion AUC_0-12_ at 4 mg was 2,563.08 PU (SD = 888.32; N = 6). A plot of the perfusion AUC_0-12_ versus concentration AUC_0-12_ demonstrated that an increase in treprostinil plasma concentrations and perfusion was observed with an increase in dose and drug exposure (Figure 4).

### Safety and tolerability

The most frequent adverse events considered probably or possibly related to the study drug following dose escalation (up to 4 mg BID, or maximally tolerated dose) of repeated twice daily doses of treprostinil diethanolamine SR were headache (17 subjects; 89%), diarrhea (nine subjects; 47%), nausea (eight subjects; 42%), fatigue (four subjects; 21%), vomiting (four subjects; 21%), flushing (three subjects; 16%) and jaw pain (three subjects; 16%). There were no serious adverse events or deaths reported during this study. The majority of adverse events were reported to be either mild or moderate in intensity. Adverse events attributed to the study drug rated as severe were headache, reported by four subjects, and nausea, jaw pain and muscle spasms, each reported by one subject. Two subjects permanently discontinued the study drug due to adverse events. One subject discontinued due to severe headache after reaching a dose of 2 mg BID. A second subject discontinued the study at 3 mg BID, following a dose reduction after exceeding the target dose and reaching 4.25 mg BID, reporting myalgia (mild), nausea (mild) and vomiting (mild). There were no clinically relevant effects on hematology, biochemistry or urinalysis parameters.

## Discussion

The primary objective of this study was to characterize treprostinil pharmacokinetics in patients with SSc following repeat doses of treprostinil diethanolamine SR up to a maximum dose of 4 mg BID. Our data show that the oral SR formulation of treprostinil diethanolamine was effectively absorbed and that improvements in skin perfusion and temperature were temporally associated with drug levels during short-term (eight-week) treatment. This study demonstrated that oral administration of treprostinil diethanolamine resulted in systemic treprostinil exposure similar to that achieved with parenteral treprostinil doses of 10 to 20 ng/kg/minute [21–24]. This is the first study to demonstrate that cutaneous perfusion may improve with an oral prostacyclin formulation. Improvements in skin perfusion and temperature were detected at the second PK visit (4 mg dose), but not the first PK visit (2 mg dose). This difference may suggest a dose-response relationship, the effect of vascular remodeling with a longer duration of treprostinil therapy, or a statistical anomaly.

Parenteral treprostinil therapy has been utilized in previous studies in SSc patients with digital ulcer disease [16–19]. Due to the delivery complexities associated with this therapy an alternative was sought to enhance patient acceptance and to improve treatment options for this population. Treprostinil diethanolamine is an innovative salt form of treprostinil that has been developed and formulated into a SR osmotic tablet oral dosage formulation to provide near zero-order release of treprostinil over the majority of a 12-hour period. In the plasma at physiological pH, treprostinil exists in the ionized state. Once ionized, treprostinil will freely associate with the predominant counter-ion in the plasma, predominantly sodium. The bioactive species derived from either salt (treprostinil sodium or treprostinil diethanolamine) exists in the same biologically active form in the plasma as treprostinil; thus, treprostinil diethanolamine is expected to retain the bioactivity and safety profile of the approved infused treprostinil sodium.

Consistent with established clinical use of prostacyclins, treprostinil diethanolamine SR requires dose titration based on signs and symptoms of disease and occurrence of adverse events. Pharmacokinetic sampling was selected at the 2 mg BID and 4 mg BID doses in this study, since they represented doses that could reasonably be achieved within an eight week escalation period. Consistent with studies in PAH patient populations, treprostinil diethanolamine SR provided plasma concentrations over the 12 hour dosing period supporting twice daily dosing in SSc patients. A linear relationship between dose and plasma concentration was observed at the 2 mg BID and 4 BID doses in SSc patients and there was no unexpected accumulation of treprostinil.

In our study following dose escalation to 2 mg BID and 4 mg BID with oral treprostinil diethanolamine SR, an increase in digital perfusion was observed with increased treprostinil blood concentrations, suggesting a dose-response relationship. After controlling for baseline perfusion, hour of assessment, and hand under study, plasma treprostinil concentration was a significant predictor of perfusion and digital skin temperature at the end of study visit. While these data show effective systemic drug absorption following oral delivery and temporally improved blood flow, further investigation with a larger sample size and a control population is needed to confirm this association, to investigate the contribution of other potentially relevant covariates to perfusion, to correct for individual subjects' natural perfusion variations, and to evaluate for dose effects.

During this study, treprostinil diethanolamine SR tablets were administered with a standardized 500 calorie meal. A recent study demonstrated that administration of treprostinil diethanolamine SR tablets following a 250 calorie meal or a 250 calorie liquid meal replacement supplement (Ensure^®^) did not have a clinically meaningful effect on the extent of treprostinil diethanolamine SR bioavailability or plasma exposure [25]. Smaller meal sizes may be required in some scleroderma patients as a result of esophago-gastric involvement of their disease, and this would not be anticipated to impact dosing in those patients. Bosentan and sildenafil are therapeutic agents used for the treatment of PAH and in Raynaud's phenomenon and digital ulcer disease in SSc [26–28]. No pharmacokinetic interaction was observed when these agents were co-administered with treprostinil diethanolamine SR [29, 30].

The results of this study demonstrated that oral therapy with treprostinil diethanolamine SR initiated at 0.25 mg BID and gradually increased in stepwise 0.25 mg BID increments was reasonably well tolerated in subjects with SSc, and the overall adverse event profile was similar to that known to be associated with prostacyclin and its analogues in other patient populations [31]. The frequency of adverse event reporting was not unexpected as investigators were instructed to continue to dose titrate in the absence of adverse events up to the target dose or a subject's maximally tolerated dose. Dose escalation was initiated in 0.25 mg BID increments every 48 hours. The titration rate was slowed at the investigator's discretion based upon individual subject tolerability. Modulation of the dose escalation rate and increasing the interval between dose increases was an effective strategy for managing adverse events. The most common adverse events reported with too fast of a titration rate or too large of a dose increase are headache, nausea, flushing, diarrhea and vomiting. Although an oral dosage form, management of treprostinil diethanolamine SR dosing closely follows experience with infused prostacyclins. Consistent with established clinical use of prostacyclins, treprostinil diethanolamine SR requires dose titration based on signs and symptoms of disease and occurrence of adverse events. Due to the large inter-patient variability in peak plasma concentration and exposure, selection of an individual dosing regimen may be required in SSc patients to titrate treprostinil diethanolamine SR to achieve clinical benefit and balance adverse effects of prostacyclins, much the same as with parenteral forms.

## Conclusions

Currently prostacyclin analogues are administered via intravenous or subcutaneous routes in scleroderma patients with vascular complications. Our data show that the oral SR formulation of treprostinil diethanolamine was effectively absorbed in patients with SSc, was associated with improved cutaneous perfusion and temperature with short-term treatment, and may provide a new therapeutic option for Raynaud's phenomenon and the peripheral vascular disease of SSc.

## Authors’ original submitted files for images

Below are the links to the authors’ original submitted files for images. Authors’ original file for figure 1 Authors’ original file for figure 2 Authors’ original file for figure 3 Authors’ original file for figure 4

## Abbreviations

AUC: area under the plasma concentration-time curve
AUC_0-12_: area under the plasma concentration-time curve from time 0 to 12 hours post dose
BID: twice daily
C_max_: maximum plasma concentration
CV%: coefficient of variation
GLSM: geometric least squares mean
LSM: least squares mean
PAH: pulmonary arterial hypertension
PK: pharmacokinetic
PU: perfusion units
QC: quality control
SR: sustained release
SSc: systemic sclerosis
t_max_: time to maximum concentration
t_1/2_: apparent terminal elimination half-life.
