# Supplementary figures and images for: Open label study of escalating doses of oral treprostinil diethanolamine in patients with systemic sclerosis and digital ischemia: pharmacokinetics and correlation with digital perfusion
Source: Arthritis Res Ther. 2013 Apr 18;15(2):R54. doi: 10.1186/ar4216 (PMC5011881; doi:10.1186/ar4216)

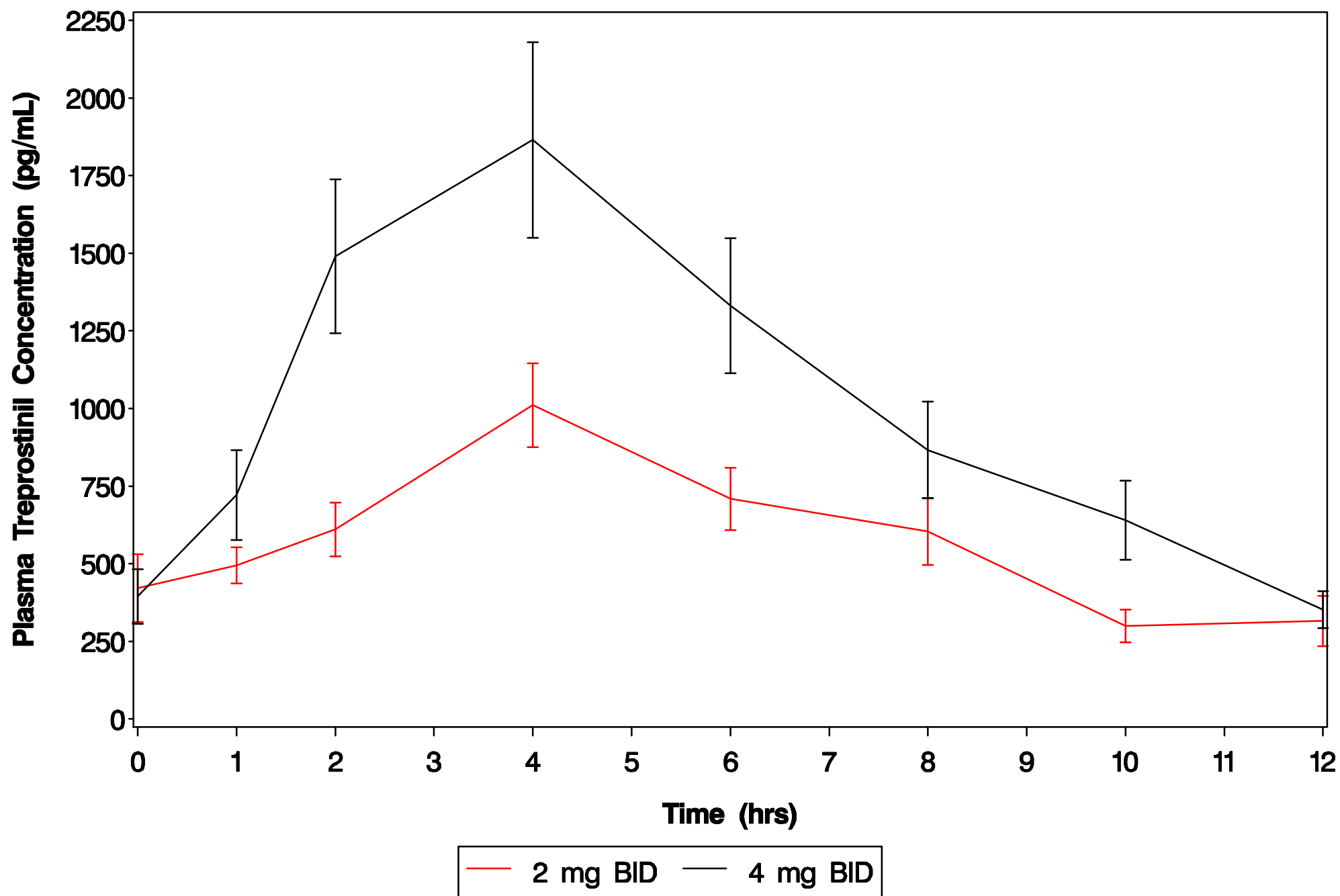

Supplement: Supplementary file 1 — Authors’ original file for figure 1 [file 13075_2012_4361_MOESM1_ESM.pdf]

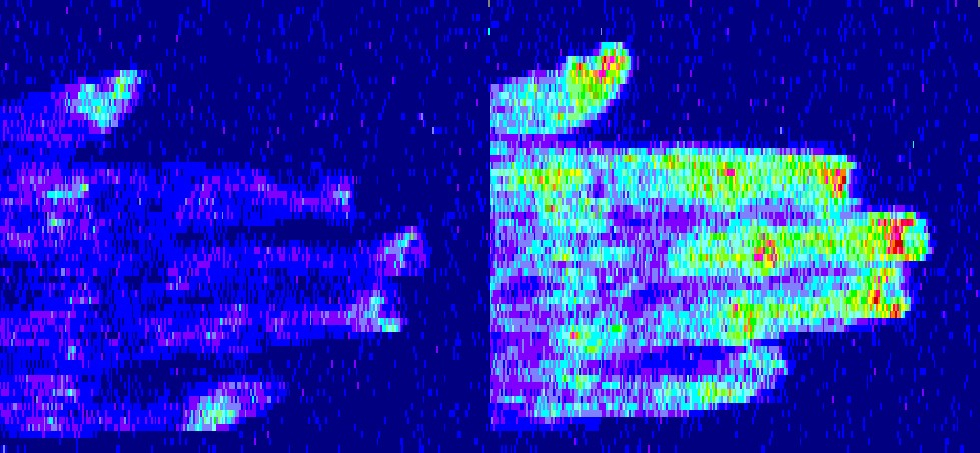

Supplement: Supplementary file 2 — Authors’ original file for figure 2 [file 13075_2012_4361_MOESM2_ESM.tiff]

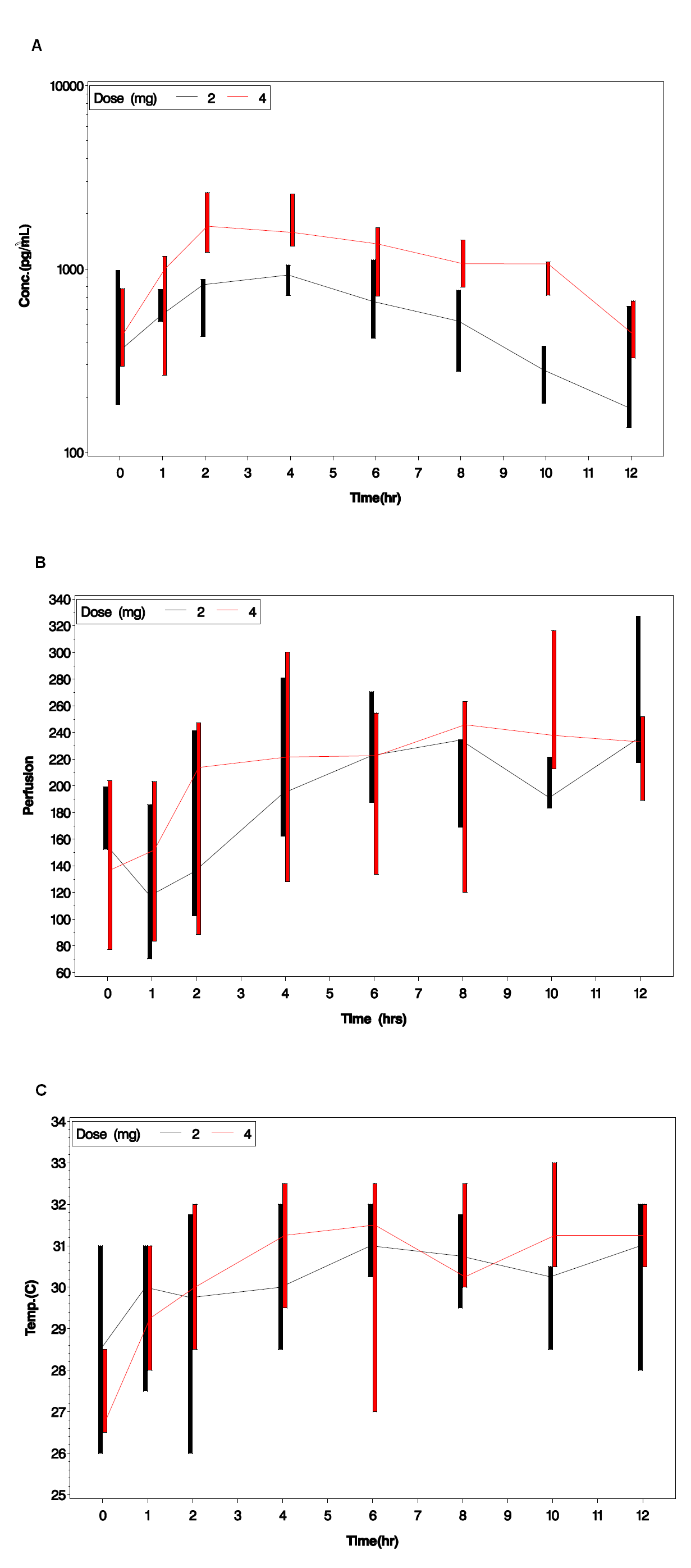

Supplement: Supplementary file 3 — Authors’ original file for figure 3 [file 13075_2012_4361_MOESM3_ESM.png]

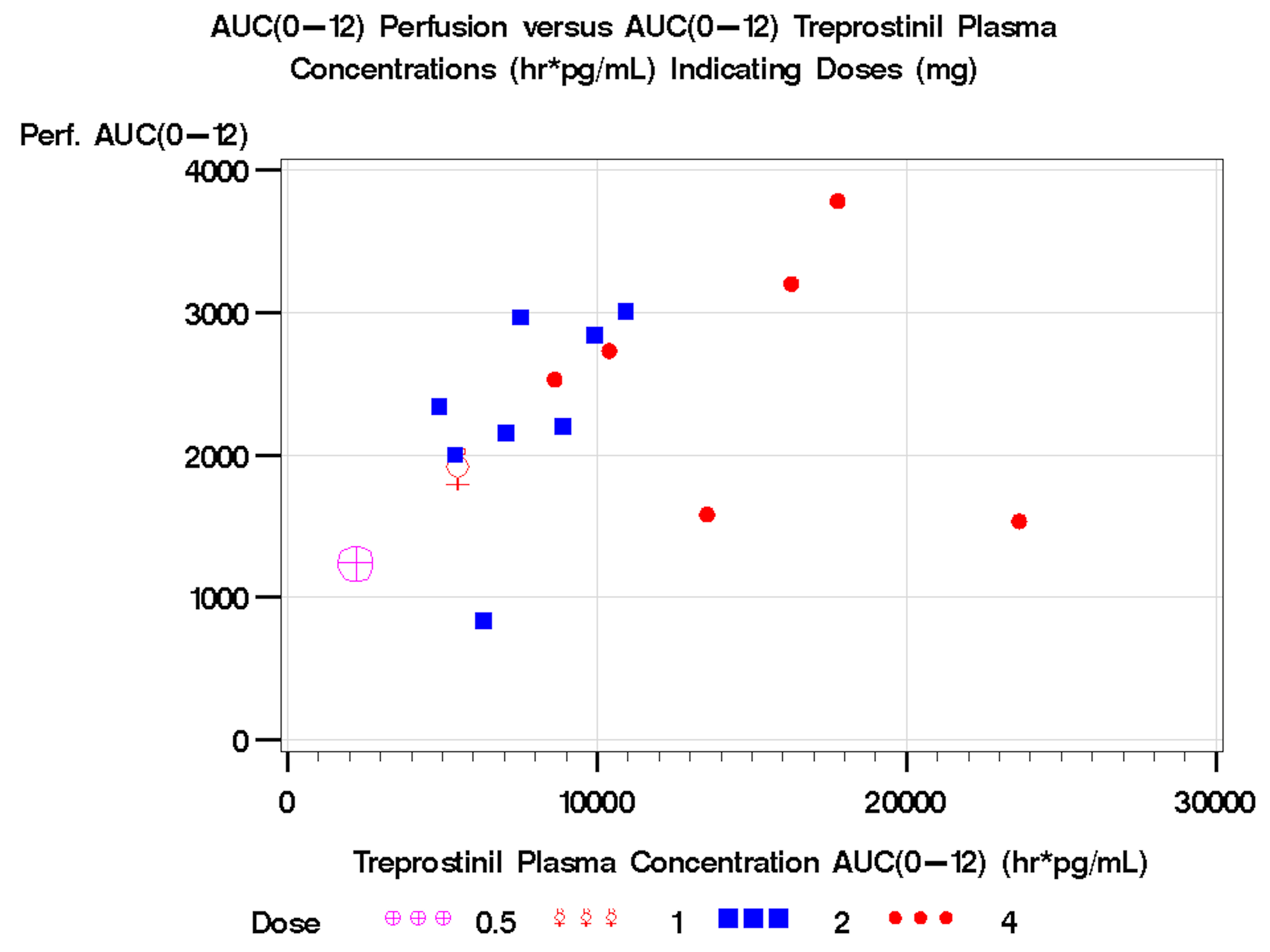

Supplement: Supplementary file 4 — Authors’ original file for figure 4 [file 13075_2012_4361_MOESM4_ESM.tiff]
